# Electronic Transport Modulation in Ultrastrained Silicon Nanowire Devices

**DOI:** 10.1021/acsami.4c05477

**Published:** 2024-06-20

**Authors:** Maximilian G. Bartmann, Sebastian Glassner, Masiar Sistani, Riccardo Rurali, Maurizia Palummo, Xavier Cartoixà, Jürgen Smoliner, Alois Lugstein

**Affiliations:** † Institute for Solid State Electronics, Technische Universität Wien, Gußhausstraße 25-25a, 1040 Vienna, Austria; ‡ Institut de Ciència de Materials de Barcelona, ICMAB−CSIC, Campus UAB, 08193 Bellaterra, Spain; § Dipartimento di Fisica and INFN, Università di Roma ”Tor Vergata”, 00133 Roma, Italy; ∥ Departament d’Enginyeria Electrònica, Universitat Autònoma de Barcelona, Bellaterra 08193, Barcelona, Spain

**Keywords:** silicon, nanowire, strain, band gap engineering, Schottky contacts

## Abstract

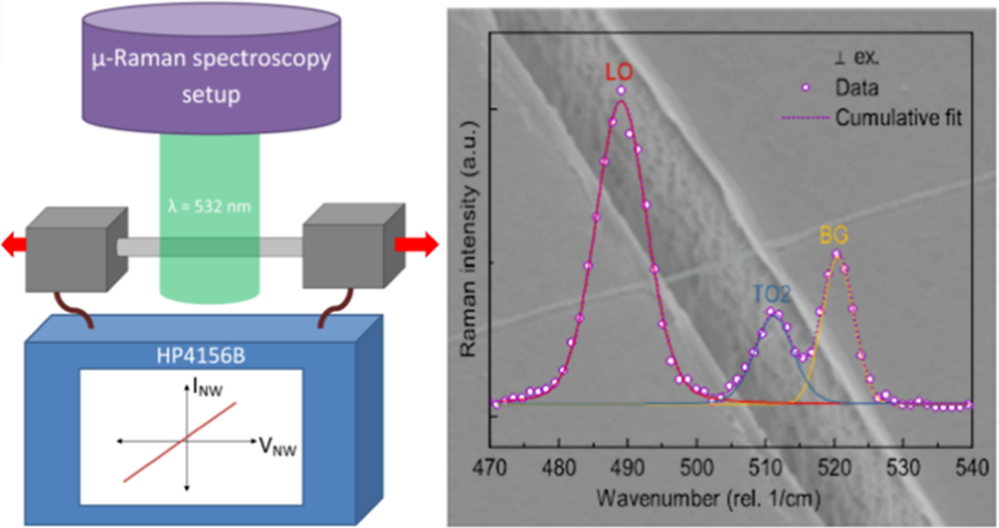

In this work, we explore the effect of ultrahigh tensile strain on electrical transport properties of silicon. By integrating vapor–liquid–solid-grown nanowires into a micromechanical straining device, we demonstrate uniaxial tensile strain levels up to 9.5%. Thereby the triply degenerated phonon dispersion relation at the Γ-point of silicon disentangle and the longitudinal phonon modes are used to precisely determine the extent of mechanical strain. Simultaneous electrical transport measurements showed a significant enhancement in the electrical conductance. Aside from considerable reduction of the Si bulk resistivity due to strain-induced band gap narrowing, comparison with quasi-particle GW calculations further reveals that the effective Schottky barrier height at the electrical contacts undergoes a substantial reduction. For these reasons, nanowire devices with ultrastrained channels may be promising candidates for future applications of high-performance silicon-based devices.

## Introduction

Strain engineering is a quite general, powerful approach that is employed to achieve significant optimization in functional materials and device performance, while retaining the native chemical composition of the material.^1−5^ Strain in semiconductors has been widely explored to alter specifically the band structure^6−8^ and thereby elementary physical properties like the effective mass,^6−9^ band alignment,^10,11^ or overall the electrical conductivity.^12^ The benefit of stress for mobility improvement has accomplished the step from basic research to industrial production of strained silicon (Si) to enhance the performance of complementary metal-oxide-semiconductor (CMOS) devices.^13^ The stringent electrostatic requirements to maintaining electrostatic integrity^14^ have thereby been met in ultrascaled transistors, where the optimum configuration is a nanowire (NW)-like architecture with a surrounding gate.^15^ Overall Si is of vital importance owing to the role it plays in nowadays semiconductor industry,^16,17^ ranging from just high-speed CMOS-integrated circuits to building blocks in nanoelectromechanical systems, biosensing,^18^ and high speed detectors.^19^

However, strain modulation is subjected to physical limits as the amount of elastic strain that can be induced in a material is limited by the maximum fracture strength. For bulk Si yield, stress lower than 3 GPa can be applied without damaging the crystal structure.^20^ Still, by reducing the dimensions, the fracture stress limits are much higher at nearly the level of Young’s modulus.^21^ Quasi-one-dimensional Si rods with a nearly defect free lattice structure can be grown via a vapor–liquid–solid (VLS)^22^ synthesis approach. This enables NWs with excellent control of the geometry and growth orientation along with superior surface conditions when compared with similar morphologies produced by common top-down methods. VLS grown Si-NWs with diameters around 200 nm have shown an increase of the fracture strain limit up to 12 GPa,^23^ attributed to their high surface-to-volume ratio, defect-scarce status, and smooth surface.^24^ Furthermore, in even thinner Si-NWs, uniaxial tensile strain values of more than 16%,^25^ close to the predicted theoretical maximum of 20% have been demonstrated without fatigue failure.^26,27^ Besides this improved stress resistance, VLS-grown NWs are also of particular interest to the semiconductor industry because their dimensions are of a technologically relevant scale, and inroads have already been made toward incorporating them into mature integrated circuit technology.^28,29^

Many research studies have been performed during the past few decades to study strain-related structural and electronic property evolution, both theoretically and experimentally. However, only a few investigations demonstrated whether ultrahigh strain can bring a new perspective in device engineering for future microelectronics and straintronics technologies.^30^ An assessment of the potential of ultrastrained NWs for high-performance device applications requires complete electrical transportation studies addressing both: (i) changes of the electronic properties such as the band gap and (ii) the behavior of the electrical contacts against uniform strain, which forms the major focus of this work.

**Figure 1:**
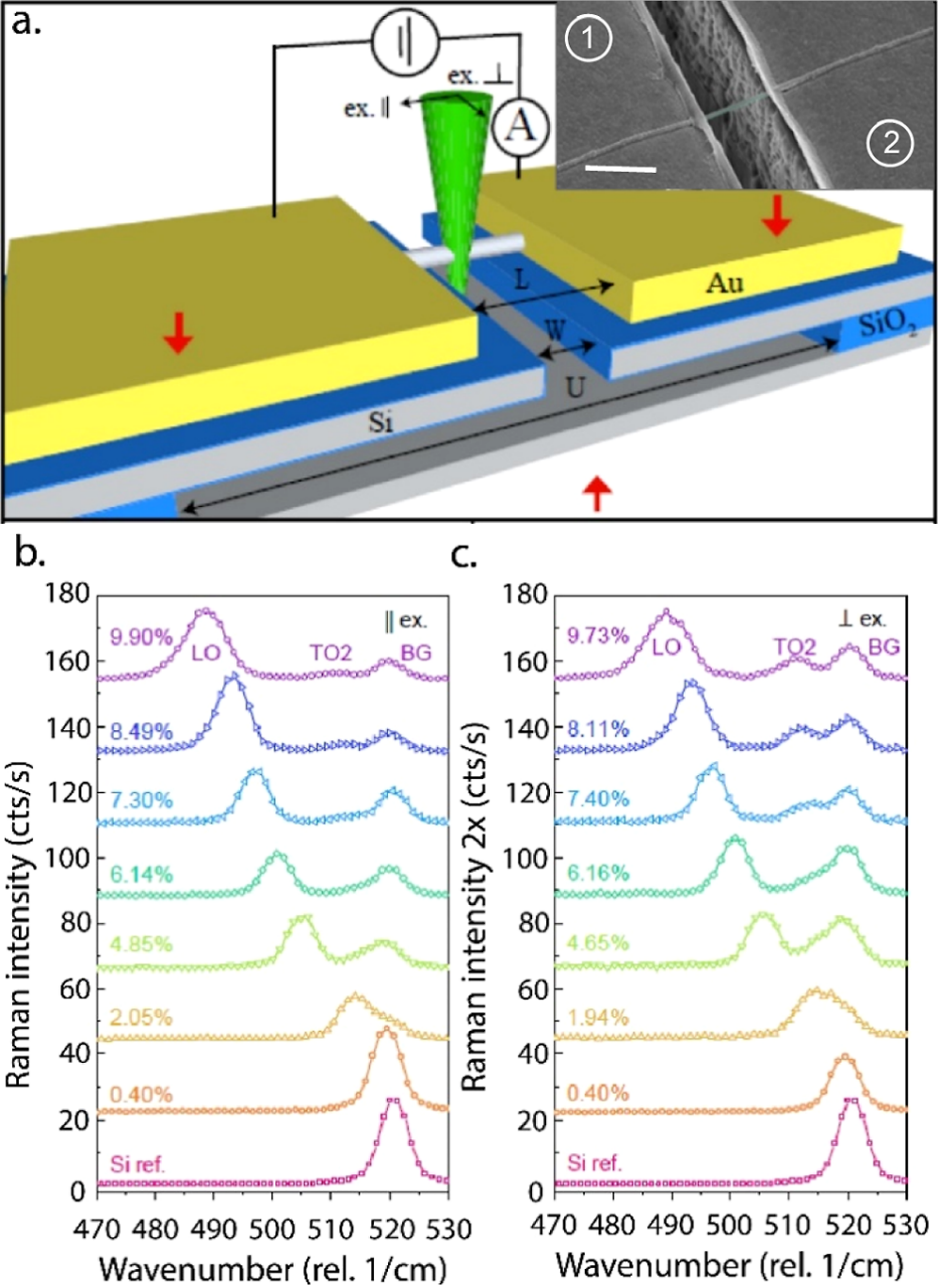
(a) Schematic illustration of the MSD with the suspended Si-NW excited by a green laser beam. Underetch, trench width, and contact gap width are indicated by *U*, *W*, and *L*, respectively. The red arrows indicate the applied forces to bend the MSD. Electrical characterization is carried out between the two Au contacts isolated from the MSD by a 100 nm thick thermally grown SiO_2_ layer. The inset shows an SEM picture of a Au contacted Si-NW with a diameter of d = 105 nm suspended over a trench with a gap width of 2 μm. Scale bar is 1 μm. (b,c) Raman spectra of the suspended NW with the laser being polarized parallel (|| ex.) or perpendicular (⊥ ex.) with regard to the NW growth axis, respectively. As the Raman signal is less intense for perpendicular laser excitation due to the dielectric mismatch, the respective signal is multiplied by a factor of 2 for better visualization.

In this work, the effect of uniaxial strain on the band gap, electrical conductivity, and the effective Schottky barrier height (SBH) at the electrical contacts of Si-NWs is investigated. To this end, we utilize a micromechanical-straining device (MSD) that allows simultaneous electrical and optical characterization of tensile-strained, VLS-grown Si-NWs. Figure 1a shows the schematic of the Si-NW on top of the MSD with gold (Au) contacts. The macroscopic Au pads fixate the NW, function as electrical contacts, and determine thus the actual length (*L*) of the strained part of the suspended NW. The two Si cantilevers of the MSD below the Au pads were formed by laser lithography and reactive ion etching from the device layer of a silicon on insulator (SOI) substrate with a gap of width (*W*). Furthermore, the buried oxide was selectively etched with hydrofluoric acid, leading to an underetch (*U*). Before NW drop casting, the whole MSD was passivated with thermally grown SiO_2_, ensuring electrical isolation of the suspended NW. Finally, the NW is fixated by contact pads via optical lithography, Au sputtering, and lift-off techniques. The inset in Figure 1a shows a scanning electron microscope (SEM) image of a ⟨111⟩ oriented VLS-grown Si-NW, integrated in the fully featured MSD. Details about the Si-NW synthesis and device integration are given in the Materials and Methods section and Supporting Information.

For the actual straining, the fully featured MSD with the Si NW was inserted in a three-point bending apparatus, as shown in the Supporting Information. Applying mechanical forces on three points, as indicated by red arrows in Figure 1a results in essentially uniaxial tensile strain along the NW, predefined by the ratio U/L.^31^ The actual strain was monitored in situ utilizing confocal μ-Raman spectroscopy with green laser excitation (λ_ex_ = 532 nm). Details about strain determination via confocal μ-Raman spectroscopy are given in the Supporting Information.

The lowermost red spectra in Figure 1b,c show the normalized Raman spectrum of the unstrained Si-NW with the exciting laser beam being polarized parallel (b) and perpendicular (c) with respect to the NW growth axis. For both polarizations, we observe a distinct first-order longitudinal optical (LO) phonon peak at 520.2 cm^–1^. Upon applying uniaxial tensile strain, the Raman peak shifts toward lower wavenumbers with a proportionality factor of about *k*_LO_ = −326, in good agreement with literature.^32^ Note that for visualization purposes, the spectrum for excitation with the laser beam being polarized perpendicular to the NW axis is magnified by a factor of 2. For perpendicular excitation of nanoscale dielectric cylinders with a diameter of *d* ≪ λ_ex_/4, the electric field inside is attenuated,^33^ yielding in an overall lower signal when compared to parallel excitation. However, Si exhibits a three-fold degenerated phonon dispersion relation at the Γ-point, which breaks under strain, resulting in the LO peak and two additional transversal optical (TO1 and TO2) peaks.^34,35^ The TO1 peak could hardly be resolved as it is superimposed by the intense LO peak. Yet, it gives rise to a distinct peak broadening at high strain levels. The TO2 peak, on the other hand, is clearly recognizable and exhibits a red-shift under tensile strain with a proportionality factor of *k*_TO2_ = −100, which is again in good agreement with theoretical predictions.^36,37^ All measurements of the strained NW show an additional small Raman peak around 520 cm^–1^, which we denote as a background (BG) peak. This peak corresponding to unstrained Si is due to inevitable excitation of the Si-based MSD as the laser beam is considerably wider than the NW.

**Figure 2:**
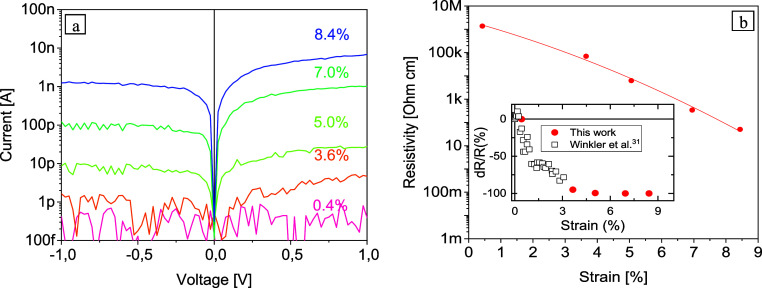
(a) Semilogarithmic plot of I/V characteristics of an ⟨111⟩ oriented VLS-grown Si-NW with a diameter *d* = 120 nm and length *L* = 2 μm under uniaxial tensile strain. (b) Strain-dependent resistivity values extracted from the *I*/*V* curves of Figure 2a. The inset shows a comparison of the relative resistivity change of this work with data from our previous highly resolved measurements however with maximum strain levels of about 3%.^38^

To characterize the overall electrical response of the strained Si-NWs, we applied voltage sweeps from −1 to +1 V and monitored the current flow at different strain levels. Only those electrical transport measurements for which subsequent Raman measurements confirmed the stability of the stress during the electrical measurement were evaluated; this appeared to be an issue for strain levels above 8%. Figure 2a illustrates typical *I*/*V* characteristics of a VLS-grown Si-NW with length *L* = 2 μm and a diameter of *d* = 120 nm for which we were able to achieve high strain levels, as well as reliable electrical measurements.

**Figure 3:**
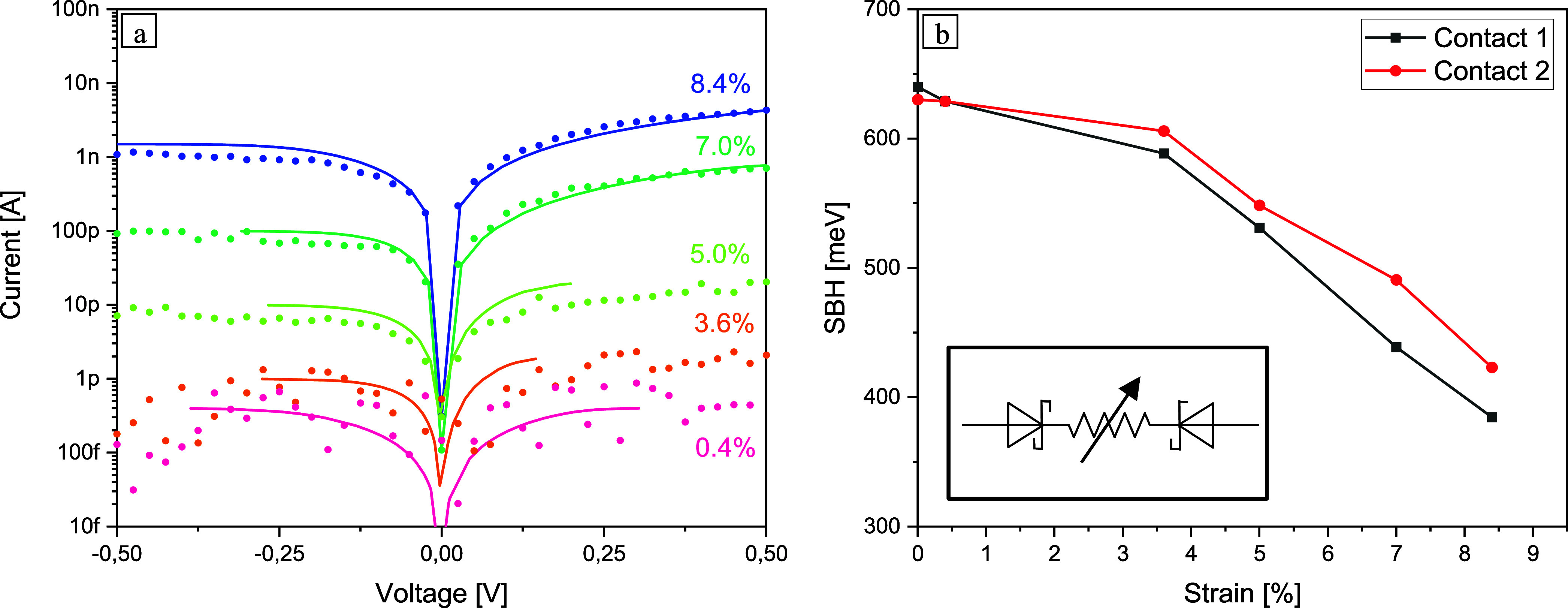
(a) Comparison of measured *I*/*V* curves (dots) of the Au contacted Si-NW at different strain levels and current–voltage characteristics constructed as *V* = *V*(*I*) according to eq 8 (solid lines). The strain-induced change of the band gap of ⟨111⟩ oriented Si-NWs was assumed to be  meV/%-strain. (b) Strain-induced lowering of the SBH of the two slightly different Au–Si contacts, as indicated in the SEM image in the inset of Figure 1a. The inset shows the circuit model used for the device simulation.

From an electrical point of view, the Si-NW integrated in the MSD could be simplified as a strain-dependent variable resistor and two Schottky contacts connected back-to-back, with one of them always polarized in the reverse direction. The respective circuit model is shown in the inset of Figure 3b. The asymmetry of the *I*/*V* characteristics in Figure 2a indicates different barrier heights and/or ideality factors of the two metal-to-semiconductor contacts most probably due to processing issues.^39^ However, unambiguously, the current rises sharply with increasing strain. Figure 2b shows thereof extracted overall decrease of resistivity of more than 4 orders of magnitude with increasing strain up to 8.4%. The inset in Figure 2b shows a comparison between the relative resistivity change of this work and the data from Winkler et al. achieved for moderate strain levels.^38^ Within this former investigations using a more sophisticated straining module with a gate wrapped around the NW,^38^ we demonstrated that for strain levels below 0.5%, stress-induced surface charge modulation resulted in an anomalous piezoresistive behavior of Si-NWs. In good agreement with Schmidt et al.,^40^ we further observed a significant decrease of the NW resistance for strain levels up to 3%, mainly due to an increased charge carrier mobility.^38^ The actual data reasonably extend the relative resistivity change of Si ⟨111⟩ NWs toward ultrahigh strain. For these, one also has to take into account strain-induced band gap narrowing resulting in an increase of the charge carrier density. Several groups reported on experimental and theoretical studies that band gap changes for Si-NWs under uniaxial tensile strain can be as large as 100 meV per 1% axial strain.^8,11,41^ Particularly for ⟨111⟩ oriented Si NWs, we determined a band gap narrowing of about 51 meV per 1% strain using quasi-particle GW calculations (Supporting Information).

To discuss how the band gap modifications affect the electrical transport, both (i) the resistance of the NW and (ii) the transport through the Schottky diodes are considered to be influenced by the applied strain. The equation for the relative change of resistivity Δϱ/ϱ for a uniaxially strained NW is given by
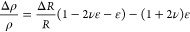
1where υ is the Poisson’s ratio and ε is the strain along the growth direction of the Si-NW. Dimensional changes are negligible as these are still very low even with this high strain and also because former work has shown that for Si, strain along the ⟨111⟩ orientation shows the smallest cross section variation.^42^

The generally accepted physical model of the current transport over a Schottky contact is thermionic emission theory where the current density is given by
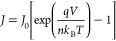
2with the saturation current density *J*_0_ given by
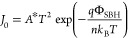
3A* is the Richardson constant, Φ_SBH_ is the effective SBH, *k*_B_ is the Boltzmann constant, *T* is the temperature, and *n* is the ideality factor. Assuming an ideal Schottky diode (*n* = 1), the current through the diode is not driven by the potential barrier for voltages higher than the barrier height but by the series resistance *R*_NW_ of the NW.^43^ The voltage drop on the Si-NW increases with increasing current and becomes the limiting factor of the current for higher voltages. Thus, the final expression for the current density through the Au contacted Si-NW is^43^
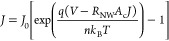
4with *A*_c_ the area of the Au contacts (∼0.7 μm^2^) determined from SEM images. Now one also has to take into account that strain-induced band gap variations have an exponential influence on the carrier concentration and in turn the resistance of the NW^44^
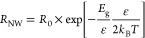
5with *R*_0_ the resistance of the unstrained NW and  meV/%-strain—the strain-induced change of the band gap of ⟨111⟩ oriented Si-NWs.

The increase in both the low-bias device conductance and the saturation current in Figure 2a suggests that not only does the NW resistance decrease but also the Schottky barriers at the electrical contacts are lowered by strain. The saturation current of a Schottky diode has an exponential dependence on the effective barrier height,^45^ which was calculated using the relation^44^
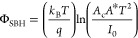
6with *I*_0_ the reverse saturation current that we obtained from the saturation region of the *I*/*V* characteristics. Due to the observed asymmetry, the current through the strained NW cannot be described by a compact expression like eq 4, but it has to take into account that the external voltage is now divided into the voltage drop along the NW and voltage drops *V*_i_ at two different Schottky contacts. Since the current flowing through the diodes is the same, the voltage drops on the respective diodes are obtained by transforming eq 2 accordingly
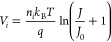
7

Adding further the series resistance R_NW_ of the Si-NW, we get the following general *V*–*I* expression
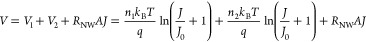
8

Since it is not possible to isolate the current as a function of voltage, *I* = *I*(*V*), Figure 3a shows the measured data and the solid lines the current–voltage characteristics constructed as *V* = *V*(*I*) according to eq 8.

The fits that we obtained exhibit excellent agreement with the experiments, even if any strain-induced change in charge carrier mobility was disregarded here. This is quite justified here as GW calculations have shown that straining levels of 8% lead to a maximum change in mobility of a factor of ∼30 (see Figure S2), i.e., much less than the overall observed change in conductivity of 4 orders of magnitude.

Strain-induced modification of the conductivity of highly strained Si-NWs can thus essentially be described sufficiently well by the following two mechanisms: (i) a reduction of the NW resistance via carrier concentration enhancement due to tensile strain-induced band gap narrowing and, accompanying this, (ii) further modification of the effective SBH. Figure 3b shows the reduction in the SBH obtained from the fit for the two slightly different Au contacts according to eq 6. Though the SBH is predicted to depend on the work-function of the metal and the electron affinity of the semiconductor,^44^ for Si most metal contacts result in Fermi level pinning^46^ close to midgap,^47^ which leads to a SBH of about half the band gap accordingly (see Figure S3). For the unstrained Si-NW with sputtered Au contacts, we determined in good agreement an effective SBH of about 600 meV and a decrease of ∼30 meV per 1% strain.

## Conclusions

We demonstrated that ultrahigh strain may play an important role in tuning the electrical properties of Si-NWs for device engineering. An MSD and a three-point bending apparatus are used to induce uniform tensile strain across ⟨111⟩ oriented Si-NWs, and simultaneous Raman measurements were used to infer and calibrate the strain on the lattice. By applying strain up to 9.7%, we were able to observe the unsetting of three-fold degenerated states in the phonon dispersion relation via confocal Raman spectroscopy. Electrical transport studies revealed the modulation of the electrical conductivity of the Si-NWs and SBH at the electrical contacts against tensile strain. In addition to the conductivity increase of the Si-NW due to strain-induced band gap narrowing, we find a reduction in the effective SBH of the Au contacts. Overall, the electrical characterization from 0 to 8.4% uniaxial strain revealed a change of the resistivity by more than 4 orders of magnitude. This effect can be used for engineering high-performance nanoscale devices. VLS-grown Si-NWs offer thus a versatile platform to apply a substantial amount of strain in a reversible manner, making it a potential platform for future microelectronics and straintronics technologies.

## Methods and Materials

Si-NWs were synthesized by means of a VLS growth technique using a low-pressure chemical vapor deposition (LPCVD) process with diluted silane as a precursor. Colloidal gold nanoparticles with a diameter of 80 nm spin coated on the hydrogen-terminated Si(111) substrates were used as catalysts for the nucleation and growth of the Si-NWs. The growth of Si-NWs was performed at 773 K using a precursor gas flow of 100 sccm (2% SiH_4_ diluted in He) and 10 sccm H_2_. The total pressure was kept at 3 mbar for 70 min. After growth, the residual gold at the NW is removed with an etching procedure consisting of a 20 s buffered hydrofluoric (BHF) acid dip to remove the native oxide followed by a 5 min aqua-regia etch step to remove the gold at the NW surface followed by another BHF dip to remove the oxide layer formed in the former etchant.

For the MSD, two Si cantilevers were formed by laser lithography and reactive ion etching from the device layer of a SOI substrate. To achieve freestanding cantilevers, the buried oxide was selectively etched with BHF acid. Before drop casting the VLS-grown Si-NWs, the whole straining device was passivated with 100 nm thermally grown SiO_2_. Finally, the NW is fixated by 200 nm thick Au contact pads via optical lithography, plasma-enhanced Au sputtering, and lift-off techniques.

Raman measurements for strain determination were performed by using a confocal μ-Raman setup (Alpha300, WITec) in back scattered geometry with a grating monochromator and CCD camera (DV401_BV, Andor). A frequency-doubled Nd:Yag laser at an excitation frequency of λ_ex_ = 532 nm is used, and the exciting laser power was limited to avoid NW heating. The laser is focused on the sample to a diffraction limited spot with a diameter of ∼500 nm. The relation between applied uniaxial tensile strain and the respective shift in the Raman spectra is given by
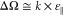
with ΔΩ representing the shift in the peak position of the Raman spectra, *k* being a proportionality factor, and ε_||_ being the strain in ⟨111⟩ growth direction of the NW. Calibration measurements comparing Raman peak shifts with physical length change of the NW determined by in situ SEM experiments revealed a proportionality factor of *k* = −326 cm^–1^.

## Supporting Information Available

NW growth, generation and calibration of strain, and calculations regarding strain-induced band gap narrowing and mobility modulation (PDF)

## Abbreviations

CMOS: complementary metal-oxide-semiconductor
NW: nanowire
VLS: vapor–liquid–solid
Si: silicon
SBH: Schottky barrier height
MSD: mechanical straining device
SOI: silicon on insulator
SEM: scanning electron microscope
LO: longitudinal optical
TO: transversal optical
