# Supplementary material for: Electronic Transport Modulation in Ultrastrained Silicon Nanowire Devices
Source: ACS Appl Mater Interfaces. 2024 Jun 20;16(26):33789–95. doi: 10.1021/acsami.4c05477 (PMC11232017; doi:10.1021/acsami.4c05477)
Supplement: Supplementary file 1 — am4c05477_si_001.pdf [file am4c05477_si_001.pdf]

Supporting Information

Electronic Transport Modulation in Ultra-strained Silicon Nanowire Devices

Maximilian G. Bartmann[1‡], Sebastian Glassner[1‡], Masiar Sistani[1‡], Riccardo Rurali[2], Maurizia Palummo[3], Xavier Cartoixà[4], Jürgen Smoliner[1], Alois Lugstein[1]*

1 Institute for Solid State Electronics, Technische Universität Wien, Gußhausstraße 25-25a, 1040 Vienna, Austria

2 Institut de Ciència de Materials de Barcelona, ICMAB--CSIC, Campus UAB, 08193 Bellaterra, Spain

3 Dipartimento di Fisica and INFN, Università di Roma Tor Vergata", 00133 Roma, Italy

4 Departament d'Enginyeria Electrònica, Universitat Autònoma de Barcelona, Bellaterra, 08193, Barcelona, Spain

Corresponding author: Alois Lugstein; email: alois.lugstein@tuwien.ac.at

Nanowire synthesis:

Si-NWs were synthesized by means of a VLS growth technique using a low pressure chemical vapour deposition (LPCVD) process with diluted silane as precursor. Prior to the deposition of the growth catalyst the Si (111) samples were cleaned with acetone and isopropanol, and blown dry with ultrahigh purity nitrogen. Subsequently the native oxide on the Si substrate was removed with a solution of buffered hydrofluoric acid (BHF; HF(38%):NH_4F =1:7) followed by a water rinse to create a hydrogen terminated surface. Colloidal gold nanoparticles with a diameter of 80 nm spin coated on the hydrogen terminated Si (111) substrates were used as catalysts for the nucleation and growth of the Si-NWs. Immediately before the samples were introduced into the hot wall LPCVD system the sample was treated once again with BHF for 10 sec. The growth of Si-NWs was performed at 773 K using a precursor gas flow of 100 sccm (2% SiH_4 diluted in He) and 10 sccm H_2. The total pressure was kept at 3 mbar for 70 min. The growth was completed by cooling down the samples in precursor gas atmosphere. After growth the residual gold at the nanowire is removed with an etching procedure consisting of a 20 sec BHF dip to remove the native oxide followed by a 5 min aqua-regia etch step to remove the gold at the nanowire surface followed by another BHF dip to remove the oxide layer formed in the former etchant.

Strain determination:

All Raman measurements were performed at room temperature. The strain at the wire was measured using a confocal μ-Raman setup (Alpha300, WITec) in back scattered geometry with a grating monochromator and CCD camera (DV401_BV, Andor). A frequency doubled Nd:Yag laser at an excitation frequency of λ_{ex} = 532 nm is used and the exciting laser power was limited to avoid a shift in the Raman spectra related to NW heating. The laser is focused on the sample to a diffraction limited spot with a diameter of ~500 nm. The relation between applied uniaxial tensile strain and the respective shift in the Raman spectra is given by:

$$\Delta\Omega \cong k * \varepsilon_{||}$$

with $\Delta\Omega$ representing the shift in the peak position of the Raman spectra, k a proportionality factor and $\varepsilon_{||}$ the strain in <111> growth direction of the nanowire. Calibration measurements comparing Raman peak shifts with physical length change of the NW reveal a proportionality factor k = -326 cm^{-1}. Therefore the length and elongation of the Si-NW under stress was

determined in situ by SEM imaging enabling a precise measurement of the NW elongation with approximately 10 nm precision without any "hidden" displacement along the electron beam direction.

Calculation of strain induced band gap narrowing in ⟨111⟩ oriented Si-NWs:

Quasi-particle calculations were carried out with the Yambo code[1,2] starting from density functional theory (DFT) wavefunctions generated with the Quantum Espresso package.[3,4] DFT calculations were performed with the Perdew-Burke-Ernzerhof (PBE) exchange-correlation functional[5] under the Generalized Gradient Approximation (GGA), with optimized norm-conserving Vanderbilt pseudopotentials.[6] Initially, a 6-atom strained cell shape (chosen because the z-axis is parallel to the [111] direction along which strain must be applied) and atomic position relaxation was carried out with a plane wave cutoff of 50 Ry, which was followed by a 2-atom self-consistent calculation with a cutoff of 30 Ry with the previously determined structure, in order to obtain the starting DFT wavefunctions. Given the large Si-NW thickness, quantum confinement can be safely neglected and calculations have assumed a bulk system. The 2-atom quasi-particle GW calculations have been performed in an 8x8x8 k-grid within a one-shot perturbative approach by using a Plasmon-Pole model[7] with 200 bands in the dielectric screening matrix and in the correlation part of the self-energy, as implemented in the Yambo code.[1,2]

Figure S1 shows the computed GW bandgap and longitudinal (*i.e.* along [111]) conduction band (CB) effective mass (m_{111}^{CB}) as a function of strain applied along the [111] direction. As strain is increased, the CB minimum shifts from a position in the Δ line towards the X point due to the X-point CB energy dropping faster than the CB minimum (strain ≤ 0.04). This causes the band to deviate from a parabolic behavior when the CB minimum and X-point energies are comparable, at the strains where no data are shown (0.04 ≤ strain ≤ 0.07), until the CB minimum is fully displaced to the X point and a new behavior of the effective mass emerges (strain ≥ 0.07). The computed GW values for the zero-strain indirect gap (1.22 eV) and m_{111}^{CB} (0.266)

show a very good agreement with the corresponding low temperature experimental values of 1.17 eV[8] and 0.259.[9]

<u>Supporting Figures:</u>

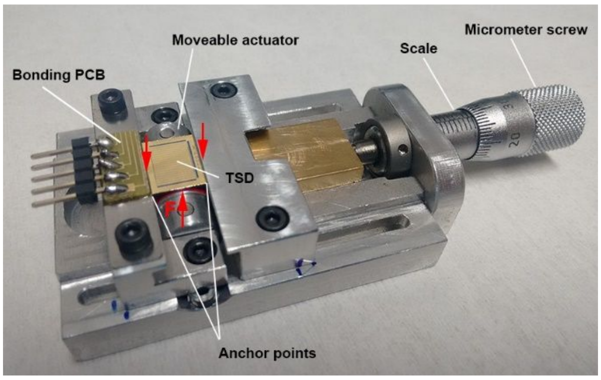

Figure S1. Optical image of the three-point bending apparatus with MSD. By turning the micrometer screw, a wedge-shaped structure is pushed under the movable actuator, which lifts the MSD chip in the middle. As the MSD is fixed at the anchor points, it is bent through. The electrical connection is established by bonding wires from the Au contact pads to the printed circuit board (PCB).

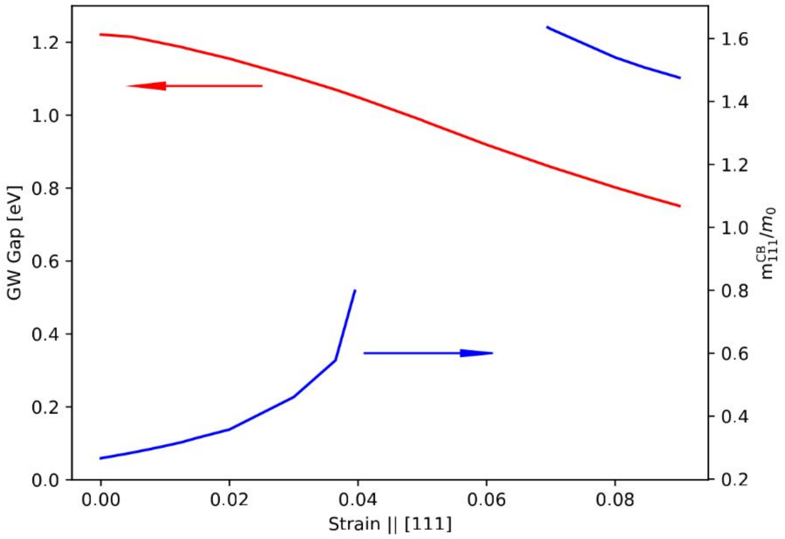

Figure S2. GW bandgap and CB effective mass along [111] *vs* strain. See text in the Supporting Information for a discussion of the missing points in the effective mass plot.

<u>Reduction of the Schottky barrier height (SBH) with strain due to Fermi level pinning:</u>

In the plot in Figure S3a the SBH is determined by the Mott rule (i.e. the difference of the metal workfunction and the semiconductor electron affinity), and since the gap reduction due to strain is mainly due to the raising of the valence band edge, the SBH remains mostly unchanged. The red line is the (constant) Fermi level, dotted lines are the intrinsic levels and the red shaded areas mean filled states at low T, with light shading meaning it is only occupied for the strained material. On the other hand, the plot in Figure S3b shows the situation with Fermi level pinning. In this case, both conduction and valence band edges shrink upon straining the material, leading to the observed SBH reduction.

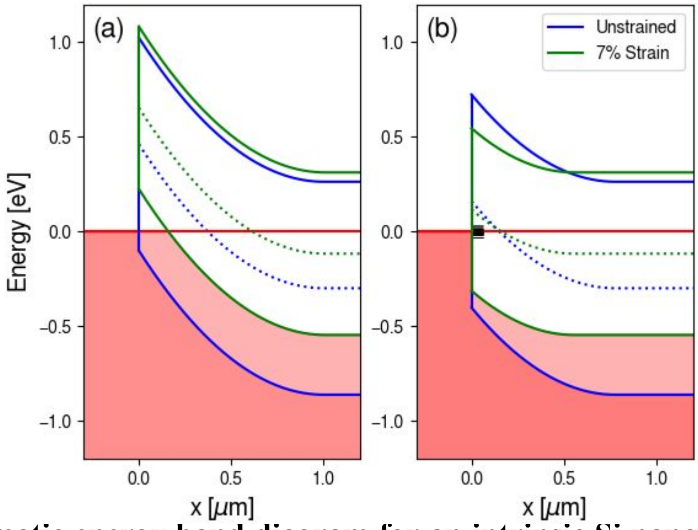

Figure S3. Schematic energy band diagram for an intrinsic Si nanowire under flat band conditions, demonstrating the influence of Fermi level pinning on effective SBH reduction due to strain induced band gap narrowing. The gap is accurate (GW) and the depletion width is calculated with the intrinsic carrier concentration taking into account the reduced gap. The position of the interface midgap states is an average of the result for the two contacts.

[1] Marini, A.; Hogan, C.; Grüning, M.; Varsano, D. Comp. Phys. Comm. 2009, 180, 1392–1403.
[2] Davide Sangalli, Andrea Ferretti, Henrique Miranda, Claudio Attaccalite, Ivan Marri, Elena Cannuccia, Pedro Miguel Melo, Margherita Marsili, Fulvio Paleari, Antimo Marrazzo, Gianluca Prandini, Pietro Bonfà, Michael O Atambo, Fabio Affinito, Maurizia Palummo, Alejandro Molina-Sanchez, Conor Hogan, Myrta Grüning, Daniele Varsano, Andrea Marini, Journal of Physics: Condensed Matter 31, 325902 (2019).
[3] P. Giannozzi, S. Baroni, N. Bonini, M. Calandra, R. Car, C. Cavazzoni, D. Ceresoli, G. L. Chiarotti, M. Cococcioni, I. Dabo, A. Dal Corso, S. Fabris, G. Fratesi, S. de Gironcoli, R. Gebauer, U. Gerstmann, C. Gougoussis, A. Kokalj, M. Lazzeri, L. Martin-Samos, N. Marzari, F. Mauri, R. Mazzarello, S. Paolini, A. Pasquarello, L. Paulatto, C. Sbraccia, S. Scandolo, G. Sclauzero, A. P. Seitsonen, A. Smogunov, P. Umari, R. M. Wentzcovitch, J. Phys.: Condens. Matter 21, 395502 (2009).
[4] P. Giannozzi, O. Andreussi, T. Brumme, O. Bunau, M. Buongiorno Nardelli, M. Calandra, R. Car, C. Cavazzoni, D. Ceresoli, M. Cococcioni, N. Colonna, I. Carnimeo, A. Dal Corso, S. de Gironcoli, P. Delugas, R. A. DiStasio Jr, A. Ferretti, A. Floris, G. Fratesi, G. Fugallo, R. Gebauer, U. Gerstmann, F. Giustino, T. Gorni, J Jia, M. Kawamura, H.-Y. Ko, A. Kokalj, E. Küçükbenli, M .Lazzeri, M. Marsili, N. Marzari, F. Mauri, N. L. Nguyen, H.-V. Nguyen, A. Otero-de-la-Roza, L. Paulatto, S. Poncé, D. Rocca, R. Sabatini, B. Santra, M. Schlipf, A. P. Seitsonen, A. Smogunov, I. Timrov, T. Thonhauser, P. Umari, N. Vast, X. Wu, S. Baroni, J. Phys.: Condens. Matter 29, 465901 (2017).
[5] Perdew, J. P.; Burke, K.; Ernzerhof, M., Phys. Rev. Lett. 1996, 77, 3865–3868.
[6] Optimized norm-conserving Vanderbilt pseudopotentials, D. R. Hamann, Phys. Rev. B 88, 085117 (2013).
[7] Godby, R. W.; Schlüter, M.; Sham, L. J., Phys. Rev. B 1988, 37, 10159-10175.
[8] W. Bludau, A. Onton, W. Heinke, J. Appl. Phys. 45 (1974) 1846.
[9] Hensel, J. C., Hasegawa, H., Nakayama, M.: Phys. Rev. 138 (1965) A225.
